# Cochlear implant in a patient with neurofibromatosis type 2 undergoing radiotherapy^☆^

**DOI:** 10.1016/j.bjorl.2015.04.004

**Published:** 2015-09-07

**Authors:** Patrícia Santos Pimentel, Danielle Seabra Ramos, Lílian Muniz, Mariana de Carvalho Leal, Silvio da Silva Caldas Neto

**Affiliations:** a Division of Otolaryngology, Hospital Agamenon Magalhães, Recife, PE, Brazil; b Human Communication Health, Universidade Federal de Pernambuco (UFPE), Recife, PE, Brazil; c Department of Speech Therapy, Universidade Federal de Pernambuco (UFPE), Recife, PE, Brazil; d Division of Otolaryngology, Universidade Federal de Pernambuco (UFPE), Recife, PE, Brazil

## Introduction

Neurofibromatosis type 2 (NF2) is a rare disease, characterized by a mutation in the long arm of chromosome 22 that leads to the development of bilateral vestibular schwannomas (VS) in approximately 90% of patients with this syndrome.^1^, ^2^, ^3^, ^4^

The control of tumor growth and hearing preservation are the cornerstones of its treatment, which can be carried out by surgery or radiotherapy, considering the size of the lesion and the presence of functional hearing.

The possibilities of hearing rehabilitation range from the adaptation of conventional hearing aids (HA) to auditory brainstem implant (ABI). In the latter case, hearing results are limited, because this procedure only enables the detection of sound and acts as an auxilliary for lip reading. Thus, several authors consider microsurgery with preservation of the cochlear nerve and use of a cochlear implant as the best therapeutic option.^3^, ^4^, ^5^, ^6^

## Case report

GMC, a 50-year-old male with progressive bilateral hearing loss for 2 years, related recent dizziness, bilateral tinnitus and headache. At physical examination, this patient revealed normal otoscopy, no nystagmus and a positive sensitized Romberg test with deviation to the right; no changes were observed in other cranial nerves.

**Figure 1:**
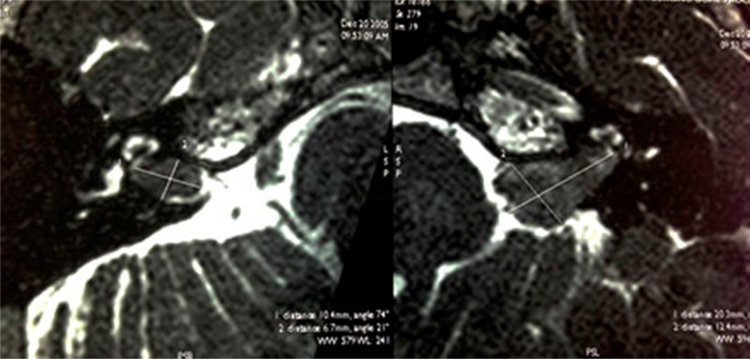
T2-weighted axial MRI before radiotherapy, showing an expansive lesion measuring 1.0 cm × 0.7 cm × 0.7 cm and occupying the right internal auditory canal (T1 according to the classification of Hannover) and another injury of the same kind measuring 2.0 cm × 1.2 cm × 0.7 cm on the left (Hannover T3a).

Audiometry revealed bilateral sensorineural hearing loss (43 dB for the right ear and >80 dB for the left ear) and the auditory brainstem evoked potential was absent in the left ear. Magnetic resonance imaging (MRI) showed solid lesions with irregular borders occupying the inside of both internal auditory canals (Fig. 1).

At that time, it was suggested left tumor surgical removal; however, given the patient's refusal, our decision was for bilateral fractionated stereotactic radiotherapy and use of hearing aids.

A year after radiotherapy, even with the tumor under control, our patient developed progression of his hearing impairment to left anacusis and severe right sensorineural hearing loss (threshold, 80 dB), with 30% discrimination of sentences in an open context using bilateral hearing aids. Thus, a right-ear cochlear implant ear was recommended, due to its smaller tumor and a residual hearing. Twelve months after implantation, the patient discriminates 100% of Ling's six-sound test, monosyllables, vowels and sentences in an open context.

## Discussion

The first cochlear implant after surgical removal of VC was reported in 1992. A decade later, we found in our search reports of 37 patients who underwent complete resection of VS, combined with a cochlear implant, with possibility of discrimination in an open context in 68% of cases.^5^

In 2013, a Belgian study related hearing preservation with radiosurgery in the treatment of NF2 in a series of 12 patients, and 78% had functional hearing (discrimination >70%) up to 2 years after radiation. However, it is important to point out that hearing impairment can happen even after several years, due to a degeneration of the stria vascularis secondary to radiation.^6^

In 2010, Trotter et al. published the results of cochlear implants in two patients with NF2 submitted to radiotherapy in the ear that received the implant. One of these patients reached 96% of discrimination of sentences, and the other, 72%. A systematic review carried out in 2012 showed that among eight patients with NF2 and deafness treated with radiosurgery, six achieved discrimination sentences in an open context with the use of a cochlear implant.^4^, ^6^

In our case, the patient with NF2 underwent fractioned stereotactic radiotherapy and a cochlear implant after tumor control; one year after implantation, this patient showed good results, being able to understand well what is spoken in a spontaneous conversation, denoting a good comprehension of speech and of its suprasegmentar traits, such as rhythm and intonation.

## Final comments

The use of a cochlear implant in patients with NF2 treated with radiotherapy is still a scarcely reported auditory rehabilitation strategy, but that has proven effective, as in the present case.

## Conflicts of interest

The authors declare no conflicts of interest.
